# Phosphorylation of BCL2 at the Ser70 site mediates RANKL-induced osteoclast precursor autophagy and osteoclastogenesis

**DOI:** 10.1186/s10020-022-00449-w

**Published:** 2022-02-19

**Authors:** Dianshan Ke, Yunlong Yu, Chenglong Li, Junyong Han, Jie Xu

**Affiliations:** 1 Shengli Clinical Medical College of Fujian Medical University, Fuzhou, 350003 Fujian China; 2 Department of Orthopedics, Fujian Provincial Hospital, No.134 Dong Jie Road, Fuzhou, 350003 Fujian China; 3 Division of Spine Surgery, Department of Orthopadics, Nanfang Hospital, Southern Medical University, Guangzhou, 510515 Guangdong China; 4 Institute for Immunology, Fujian Academy of Medical Sciences, Fuzhou, 350003 Fujian China

**Keywords:** BCL2 phosphorylation, Ser70, Ser87, RANKL, Osteoclast, Autophagy

## Abstract

**Background:**

Phosphorylation modification of BCL2 is involved in receptor activator of nuclear factor-κB ligand (RANKL)-induced autophagy of osteoclast precursors (OCPs) and osteoclastogenesis. As an antiapoptotic molecule, the role of BCL2 phosphorylation in osteoclastogenesis is unknown. This study aimed to explore how BCL2 phosphorylation at specific sites regulates osteoclastogenesis.

**Methods:**

We first examined the effects of RANKL on BCL2 phosphorylation at different sites (Ser70 and Ser87) in OCPs. In vivo, transgenic mice overexpressing RANKL (Tg-hRANKL mice) were used to observe the effects of RANKL on phosphorylated BCL2 at different sites in OCPs of trabecular bone. Subsequently, using site-directed mutagenesis, we observed the respective effect of BCL2 mutations at different phosphorylation sites in OCPs on osteoclastogenesis, apoptosis, autophagy and the affinity between BCL2 and Beclin1/BAX under RANKL intervention.

**Results:**

RANKL promoted BCL2 phosphorylation at the Ser70 (S70) site, but not the Ser87 (S87) site, in OCPs. Moreover, Tg-hRANKL mice had stronger BCL2 phosphorylation capacity at S70, not S87, in the OCPs of trabecular bone than wild-type mice in the same nest. Furthermore, BCL2 mutation at S70, not S87, inhibited RANKL-induced osteoclast differentiation and bone resorption activity. In addition, BCL2 mutation at S70 promoted OCP apoptosis, while BCL2 mutation at S87 showed the opposite effect. Remarkably, the BCL2 mutation at S70, not S87, inhibited OCP autophagic activity. Furthermore, BCL2 mutation at S70 enhanced the coimmunoprecipitation of BCL2 and Beclin1, whereas BCL2 mutation at S87 enhanced the coimmunoprecipitation of BCL2 and BAX in OCPs. More importantly, OCP autophagy, osteoclast differentiation and resorption pits inhibited by BCL2 mutation at S70 could be reversed by Beclin1 upregulation with TAT-Beclin1.

**Conclusion:**

RANKL activates OCP autophagy through BCL2 phosphorylation at S70, thereby promoting osteoclastogenesis, which indicates that the inactivation of BCL2 at S70 in OCPs may be a therapeutic strategy for pathological bone loss.

**Supplementary Information:**

The online version contains supplementary material available at 10.1186/s10020-022-00449-w.

## Introduction

Pathological bone loss is a group of diseases related to the skeletal system based on the imbalance between osteoblast-mediated bone formation and osteoclast-mediated bone resorption, which is characterized by decrease bone mass and damaged bone microstructure (Yu et al. 2021; Fujii et al. 2021). The enhancement of bone resorption activity induced by the pro-osteoclastogenic factor RANKL is the main pathological basis (Boyle et al. 2003; Xiong et al. 2018; Yang et al. 2021). RANKL is involved in a variety of pathological bone losses (Eghbali-Fatourechi et al. 2003; Van Tuyl et al. 2010; Roux and Mariette 2010; Raje et al. 2019). Dinosumab, a monoclonal antibody against RANKL, has been used in the clinical treatment of the above diseases (Cummings et al. 2009; Raje et al. 2018; Takeuchi et al. 2019; Chawla et al. 2019). A better understanding of the mechanism of RANKL during osteoclastogenesis and finding more efficient and specific therapeutic targets are the keys to the prevention and treatment of pathological bone loss.

RANKL can induce osteoclastogenesis through JNK1 signalling (David et al. 2002; Ikeda et al. 2004). In addition to the identified c-Jun/AP-1 pathway, there is also a BCL2-Beclin1-autophagy activation pathway in JNK1-mediated osteoclastogenesis (Ke et al. 2019a). JNK1 causes the dissociation of the B-cell lymphoma 2 (BCL2)-Beclin1 complex by the phosphorylation of BCL2, further inducing autophagy activation (Wei et al. 2008a), which is also found in RANKL-induced osteoclastogenesis (Ke et al. 2019a). Protective autophagy is involved in the differentiation, survival protection, proliferation and bone resorption activity of osteoclasts (DeSelm et al. 2011; Lin et al. 2016; Ke et al. 2019a; Cheng et al. 2020). Importantly, Beclin1, an autophagy regulatory molecule, is indispensable in osteoclastogenesis (Chung et al. 2014; Xiu et al. 2014; Arai et al. 2019; Ke et al. 2019a). However, as a positive factor of Beclin1-dependent autophagy, the role of BCL2 phosphorylation in osteoclastogenesis remains unclear.

It should be noted that BCL2 phosphorylation not only activates autophagy but also promotes apoptosis (Wei et al. 2008b). As a representative antiapoptotic molecule, BCL2 can specifically bind with BAX and other pro-apoptotic proteins to prevent the permeabilization of the mitochondrial membrane, thus resisting apoptosis (Levine et al. 2008). BCL2 phosphorylation promotes the release of BAX from the BCL2-BAX complex, thereby promoting apoptosis (Bassik et al. 2004; Wei et al. 2008a). A previous study showed that overexpression of BCL2 in transgenic mice can enlarge the volumes of osteoclasts (Bozec et al. 2008). Consistently, BCL2-knockout mice demonstrated impaired osteoclast formation and activity in vivo (Yamashita et al. 2008). Unexpectedly, the osteoclastic induction of OCPs in BCL2-knockout mice resulted in a significant increase in the number and volume of osteoclasts (Yamashita et al. 2008). It is inferred that BCL2 plays more than just a positive role in osteoclastogenesis. Accordingly, the direct inhibition of BCL2 may reduce the targeting and effectiveness of treatment. However, BCL2 phosphorylation exerts a pivotal effect on its regulation of apoptosis (Yang and Chan 2009), which is of great research significance. BCL2 phosphorylation at Ser70 maintains the antiapoptotic effect of BCL2, whereas phosphorylation at Ser87 inhibits the antiapoptotic effect (Liu et al. 2010; Liu et al. 2018; Saatci et al. 2018). As a mechanism to protect cells, autophagy has antiapoptotic effects (Fitzwalter and Thorburn 2015; Fitzwalter et al. 2018; Wang et al. 2020; Young et al. 2020). Moreover, autophagy induced by RANKL can resist OCP apoptosis (Ke et al. 2019a; b). Therefore, it is feasible to elucidate the role of BCL2 in osteoclastogenesis through a phosphorylation modification mechanism. We hypothesize that BCL2 phosphorylation at different sites exerts different effects on OCP survival and osteoclast formation. However, under the action of RANKL, BCL2 phosphorylation at the corresponding site is likely to mediate protective autophagy, subsequently promoting osteoclastogenesis through effective functions including apoptosis resistance.

Based on the above conjecture, two amino acid sites on BCL2, namely, Ser70 and Ser87, were selected as research targets. However, under RANKL intervention, the exact site of BCL2 phosphorylation in OCPs needs to be determined. The specific roles of BCL2 phosphorylation at corresponding sites in osteoclastogenesis are also unknown. In addition, the specific roles of BCL2 phosphorylation at different sites in OCP autophagy, apoptosis and the underlying mechanisms require further exploration.

## Materials and methods

### Animals

4 ~ 8-weeks-old C57BL/6 female mice and 7-weeks-old Tg-hRANKL mice (20–25 g) were obtained from the Animal center of Gem Pharmatech Co., Ltd (Nanjing, China). The animals were housed in a common environment in which the room temperature was 20 ~ 30 °C and the humidity was 60 ~ 80%, and were fed a general laboratory diet.

### Extraction and induction of OCPs

The tibiae from 4 to  8-week-old C57BL/6 mice were flushed with alfa-Minimum Essential Medium (α-MEM) without serum. The bone marrow cells were incubated with α-MEM supplementing 10% fetal bovine serum (FBS), penicillin (100 U/mL) and streptomycin (100 mg/mL) for 24 h. Non-adherent cells were harvested as bone marrow-derived macrophages (BMMs). BMMs were induced as OCPs under the intervention of M-CSF (30 ng/mL in all experiments; Peprotech, NJ, USA) for 3 days as previously described (Lu et al. 2009; Ha et al. 2010). The cells were incubated in the humidified atmosphere with 5% CO_2_ and 37 °C. FBS concentration added to the cell culture medium varies according to different experiments.

### Osteoclast differentiation assay

Cells (8 × 10^4^ cells/well) were incubated in 24-well plates and treated with M-CSF plus RANKL (100 ng/mL in all experiments; R&D Systems, MN, USA) for 4 days. Osteoclast differentiation level was determined by Tartrate resistant acid phosphatase (TRAP) staining using the relevant kit (Sigma-Aldrich, MO, USA) according to manufacturer’s protocols. TRAP-positive cells having more than 3 nuclei were defined as mature osteoclasts. TRAP-positive cells having more than 5 nuclei were defined as large osteoclasts.

### Bone resorption assay of osteoclasts

OCPs were stimulated using M-CSF plus RANKL for 2 days, isolated and re-seeded onto sterile bone discs (Corning, NY, USA), and adhered to the surface for 6 h before reagent treatment. These cells were then subjected to different treatments for another 6 days. Cells were removed by ultrasound and stirring, and the resorption pits were imaged under a scanning electron microscope (FEI Quanta 250, Thermo Fisher Scientific). Bone resorption areas were evaluated via ImageJ software (version 1.8.0).

### Site-directed mutagenesis of OCPs

Mouse BCL2 cDNA was cloned in pUC19 plasmid. The nucleotides related to each serine (s) residue were replaced with corresponding nucleotides to cause a conserved alteration to alanine (A) using a site-directed mutagenesis Kit (Clontech, San Francisco, CA, USA). Each mutation was identified by cDNA sequencing and then cloned into pCIneo mammalian expression vector (Promega, Madison, WS, USA). pCIneo plasmid containing each BCL2-mutating cDNA was transfected into M-CSF-pretreated OCPs using electroporation. The efficiency of site-directed mutagenesis was verified using western blot analyses.

### Western blotting assays

The lysates from OCPs (2 × 10^6^ cells/well) with the indicated treatments were prepared from 6-well plates. Cell lysates were packed into 10% SDS-PAGE gels and transferred to polyvinylidene fluoride membranes (PVDF). Subsequently, the PVDF membranes were incubated with antibodies targeting phosphorylated BCL2 (p-BCL2, S70, 1:1000), phosphorylated BCL2 (p-BCL2, S87, 1:1000), BCL2 (1:1000) (Thermo Fisher Scientific, MA, USA), Cathepsin K (CTSK, 1:1000), matrix metallopeptidase 9 (MMP9, 1:1000), TRAP (1:2000), Cleaved-caspase3 (1:5000), PARP (1:1000), LC3B (1:2000), p62 (1:10,000), Tubulin (Abcam, Cambridge, UK). Horseradish peroxidase (HRP)-linked secondary antibodies were applied as the secondary antibodies. The signals were visualized using a chemiluminescence system (Amersham Image 600, General Electric, MA, USA).

### Coimmunoprecipitation assay

The total protein was extracted by RIPA Lysis and Extraction Buffer (Thermo Fisher Scientific). Next, we rinsed the beads with 100 μL iced buffer, added 100 μL antibody-binding buffer to revolve the antibody and magnetic beads for 30 min, and then rinsed the beads 3 times using 200 μL buffer for 5 min each time. Cell lysates and antibody-bound magnetic beads were incubated for 1 h at room temperature and washed using 200 μL buffer for 5 min each time. 20 μL eluent was used to rinse the beads once and the supernatant was removed. The cell lysates were extracted for Co-IP with anti-BCL2 (1:100), Beclin1 (1:100), BAX (1:100) antibodies (Thermo Fisher Scientific), and then, precipitates were detected using western blotting with anti-Beclin1 (1:1000), BAX (1:1000), BCL2 (1:1000) antibodies, respectively.

### Detection of autolysosomes

The formation of autolysosomes was identified by transmission electron microscopy (TEM) assays. After the indicated treatments, the preparation, fixation, dehydration, infiltration, baking, slicing and staining of samples were performed as described previously (Ke et al. 2019a). The sections were observed under the 7700 TEM (Hitachi, Tokyo, Japan).

### Animal experiment

After breeding, 3-months-old male Tg-hRANKL mice and littermate wild-type mice were used in the experiment (N = 6, per group). Firstly, their genotypes were identified by PCR assays. Next, all mice were sacrificed. The tibiae were harvested, wrapped in 0.9% saline-soaked gauze and stored at − 20 °C. The experimental protocols were approved by the Animal Ethics Committee of Fujian Provincial Hospital (2016-0004).

### Micro-computed tomography (Micro-CT)

Three-dimensional (3D) reconstructions of the cancellous bones in the proximal tibia metaphysis were made using Bruker Micro-CT Skyscan 1276 system (Kontich, Belgium). Scan settings are as follows: voxel size 6.533481 μm, medium resolution, 55 kV, 200 mA, 1 mm Al filter and integration time 384 ms. Bone density measurements were calibrated to the manufacturer’s calcium hydroxyapatite (CaHA) phantom. Analysis was performed using the manufacturer’s evaluation software. Three-dimensional (3D) reconstruction was accomplished by NRecon (version 1.7.4.2). The parameters included Bone Mineral Density (BMD), Bone Volume/Tissue Volume (BV/TV), Bone Surface/Bone Volume (BS/BV), Trabecular Thickness (Tb.Th), Trabecular Number (Tb.N) and Trabecular Separation (Tb.Sp) (N = 6, per group).

### Haematoxylin and eosin (H&E), TRAP staining and immunofluorescence (IF) assays in tissues

The tibiae of all mice were fixed in 4% paraformaldehyde (PFA) for 48 h and decalcified in 10% EDTA (pH 7.3) for 2 weeks at 4 °C. Then, all samples were dehydrated in graded ethanol series and embedded in paraffin (Leica). All section (5 μm thick) were stained with the H&E or TRAP staining kit (N = 6, per group). The trabecular bone area (%Tb.Ar) of the H&E-stained sections was analysed using Image-Pro Plus (IPP, version 7.0) software. The osteoclast number in the TRAP-stained sections were assessed via an eyepiece grid. For IF (p-BCL2-S70, 1:200, Thermo Fisher Scientific; p-BCL2-S87, 1:200, Thermo Fisher Scientific; RANK, 1:500, Santa Cruz Biotechnology), all sections were incubated in citrate buffer (10 mM citric acid, pH 6.0) at 60 °C overnight to expose the antigens. We incubated the primary antibodies at 4 °C overnight and the secondary antibodies at room temperature for 1 h (N = 6, per group). The fluorescent intensity of p-BCL2 at the corresponding sites in RANK^+^ cells was evaluated using IPP software and calculated as the ratio of RANK^+^ p-BCL2^+^ cells to RANK^+^ cells.

### Fluorescence-activated cell sorting (FACS) of isolated bone marrow RANK^+^ CSF1R^+^ cells

Bone marrow cells were isolated from each Tg-hRANKL mouse and corresponding control mouse. 70 micron-nylon mesh was used to disperse cells into single-cell suspensions, rinsed and resuspended in 5 mL ice DMEM for 3 min. Nonspecific binding was blocked by treated cells with rat anti-mouse CD16/32 monoclonal antibody (TruStain FcX™ PLUS, BioLegend, CA, USA) at room temperature for 15 min. Cells were stained using PE anti-mouse RANK and CSF1R antibodies, and the cells were harvested on a MoFlo XDP flow cytometer (Beckman Coulter). The harvested RANK^+^ CSF1R^+^ cells were used for the immunofluorescence assay.

### Cellular immunofluorescence assays

The enriched RANK^+^ CSF1R^+^ cells (1 × 10^6^/well) were seeded on 6-cm dishes and fixed using 4% PFA. After perforation, the cells were blocked using 1% bovine serum albumin (BSA) and incubated with anti-p-BCL2 (S70, 1:200) or p-BCL2 (S87, 1:200) antibodies (Thermo Fisher Scientific) at 4 °C overnight. Next, the cells were stained with fluorochrome-labelled secondary antibody for 30 min, and subsequently counterstained with DAPI for 10 min. Finally, the stained cells were observed and recorded using fluorescence microscopy (Olympus IX81, Tokyo, Japan).

### Detection of apoptosis

Apoptosis levels were assessed through Annexin V–FITC/PI staining and detection of apoptotic protein. After the specific treatment in the experimental design, the indicated cells were harvested, and then staining was carried out in accordance with manufacturer’s protocols. Next, the apoptosis levels were measured and quantitatively analysed using the flow cytometer (BD Accuri C6 Plus, BD Biosciences, NJ, USA). Apoptotic proteins were detected by western blotting.

### Detection of mitochondrial membrane potential

The mitochondrial membrane potential was detected using JC-10 assays kit (tetraethyl benzimidazolylcarbocyanide iodine, Sigma-Aldrich, MO, USA). The treated OCPs (20,000 cells/well) were plated in a microplate. The cells were stained with JC-10 solution according to manufacturer’s protocols. The accumulation of JC-10 dye is proportional to mitochondrial membrane potential. The cells were added with about 100 µL JC-10 dye and incubated for 20 min. The alteration in fuorescence intensity was measured using a microplate reader (Molecular Devices, Sunnyvale, CA, USA) at λex = 490/λem = 525 nm and λex = 540/λem = 590 nm. The mitochondrial membrane potential levels were determined by measuring the ratio of red/green fluorescence intensity and quantitatively analysed using the flow cytometer (BD Accuri C6 Plus).

### Statistical analyses

Data are presented as the mean ± SEM. Statistical analyses were performed using one-way ANOVA and Student’s T Test. The P value was set at 0.05. All statistical analyses were carried out by the SPSS 19.0 software.

## Results

### RANKL promoted BCL2 phosphorylation at the S70 site, but not the S87 site, in OCPs

**Fig. 1:**
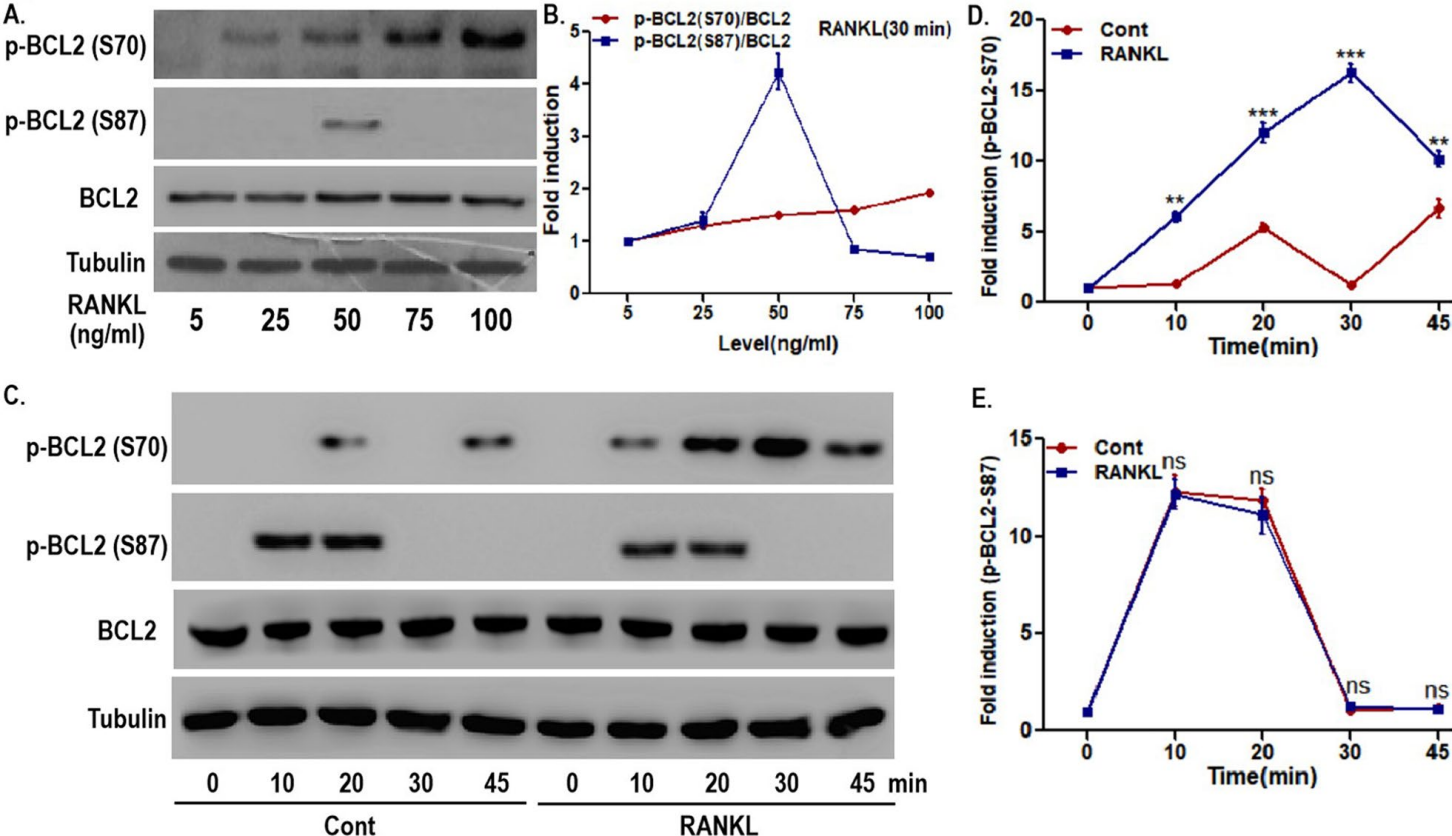
RANKL promoted BCL2 phosphorylation at S70, not S87, in OCPs. **A** OCPs were treated with the indicated concentration of RANKL for 30 min in α-MEM with 1% FBS (appropriate starvation for enhancing the phosphorylation effect). Phosphorylated BCL2 at S70, i.e., p-BCL2 (S70), and phosphorylated BCL2 at S87, i.e., p-BCL2 (S87), were detected using western blotting assays. **B** Dynamic changes in p-BCL2 (S70) and p-BCL2 (S87) in OCPs. The protein expression level was normalized to that of the control samples (5 ng/mL). **C** OCPs were treated with or without 100 ng/mL RANKL for the indicated times in α-MEM with 1% FBS. p-BCL2 (S70) and p-BCL2 (S87) were detected using western blotting assays. **D**, **E** Dynamic changes in p-BCL2 (S70) and p-BCL2 (S87) in OCPs. The protein expression level was normalized to that of the control samples (0 min). At different time points, pairwise comparisons were made between the RANKL group and the control group. The experiments were replicated at least three times. Data are presented as the mean ± SEM from three independent experiments. ***P < 0.001, **P < 0.01. *ns* not significant, *Cont* the control group without RANKL intervention

First, the effect of RANKL on two phosphorylation sites of BCL2 in OCPs was identified. As shown in Fig. 1A, B, under RANKL intervention, the expression of phosphorylated BCL2 at the S70 site increased in a concentration-dependent manner in OCPs. However, the level of phosphorylated BCL2 at the S87 site decreased significantly as the working concentration of RANKL reached 50 ng/mL (Fig. 1A, B). In addition, the time variation curve of S70-phosphorylated BCL2 in OCPs with RANKL intervention was significantly higher than that in OCPs without RANKL intervention (Fig. 1C–E). Nevertheless, there was no significant difference in the time curve of S87-phosphorylated BCL2 between the RANKL group and the control group (Fig. 1C–E). Moreover, the expression of S70-phosphorylated BCL2 in the RANKL group was higher than that in the control group from 10 to 45 min (Fig. 1C, D). However, compared with the control group, the RANKL group had no stronger phosphorylation of BCL2 on S87 (Fig. 1C, E). These results suggest that RANKL could significantly phosphorylate BCL2 at the S70 site in OCPs. In contrast, RANKL did not affect BCL2 phosphorylation at the S87 site in OCPs.

### Tg-hRANKL mice had stronger BCL2 phosphorylation capacity at S70, not S87, in OCPs

**Fig. 2:**
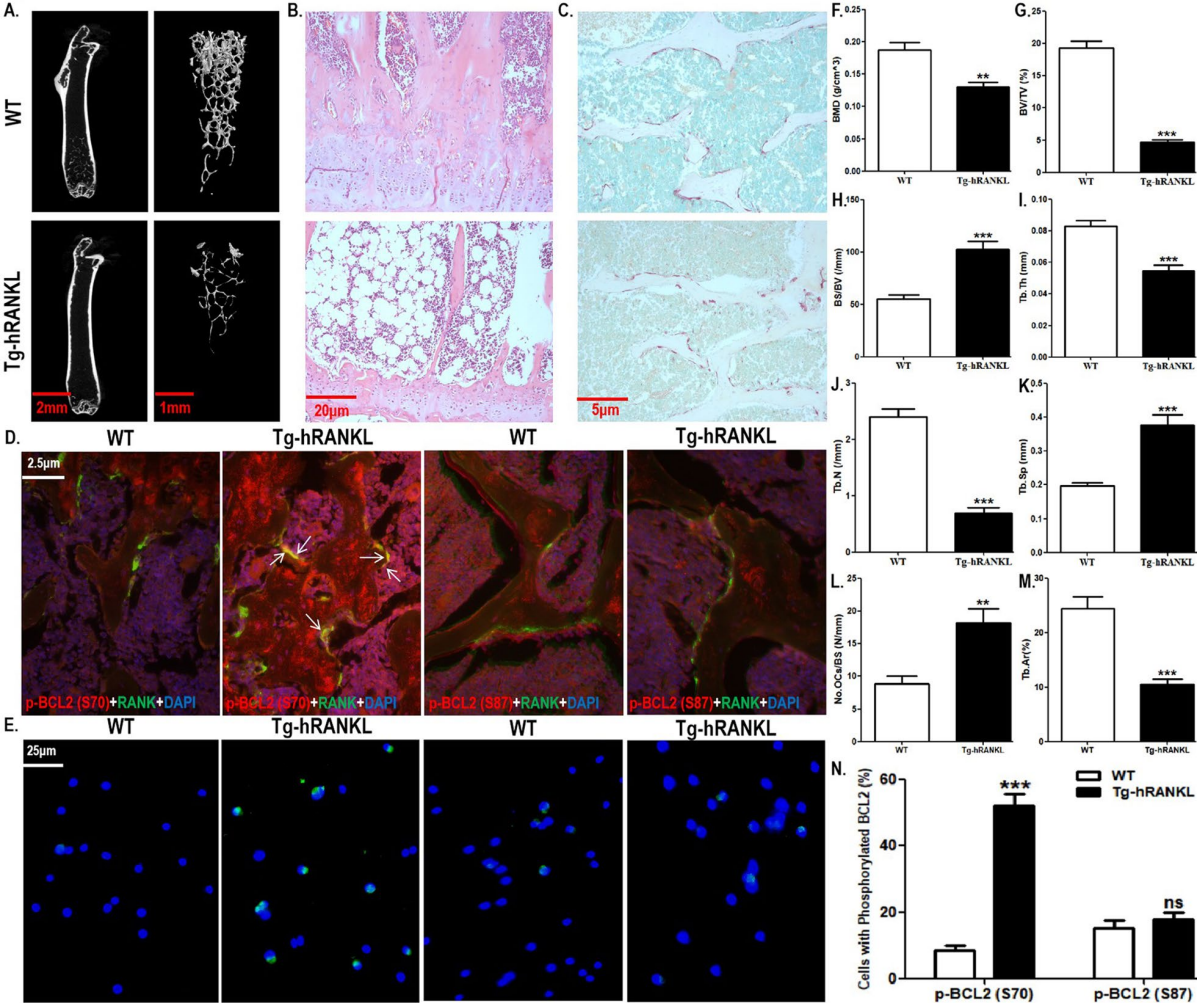
Tg-hRANKL mice had stronger BCL2 phosphorylation capacity at S70, not S87, in OCPs. **A** Representative 3D micro-CT reconstructed images of the tibiae from Tg-hRANKL mice and control mice in the same nest (WT mice) showing bone mass and bone microstructure (N = 6/group). Scale bar, 2 mm or 1 mm. **B** Representative H&E-stained tibial sections from each group. Scale bar, 20 μm. **C** Representative TRAP-stained tibial sections from each group (Red arrows indicate TRAP^+^ cells). Scale bar, 5 μm. **D** Tibial sections were stained with red and green fluorochromes for p-BCL2 (S70 or S87) and RANK, respectively, and observed using fluorescence microscopy. The overlapping staining of p-BCL2 and RANK are indicated with red arrows (yellow fluorescence). Scale bar, 2.5 μm. **E** Representative fluorescent images of p-BCL2 (S70 or S87) (red arrows) in bone marrow RANK^+^ CSF1R^+^ cells sorted by FACS. Scale bar, 20 μm. **F**–**K** The trabecular bone parameters, including BMD, BV/TV, BS/BV, Tb.Th, Tb.N, and Tb.Sp were analysed using micro-CT. **L** The trabecular bone parameter, Tb.Ar, was analysed using H&E staining and IPP system. **M** The number of osteoclasts per millimeter of trabecular bone surface was counted. (N) The percentages of p-BCL2 (S70)-positive cells or p-BCL2 (S87)-positive cells in **E** (30 cells per field, N = 5). The experiments were replicated at least three times. Data are presented as the mean ± SEM. ***P < 0.001, **P < 0.01. *ns* not significant, *WT* control mice in the same nest

We documented the differential effects of RANKL on BCL2 phosphorylation at different sites in OCPs in vitro. Next, we used Tg-hRANKL transgenic mice to observe the in vivo effect of RANKL on BCL2 phosphorylation at different sites in OCPs. Tg-hRANKL mice have been used as a typical animal model of osteoporosis due to overexpressed RANKL (Rinotas et al. 2014; Bonnet et al. 2019). Here, although there was no significant difference in the appearance between Tg-hRANKL mice and control mice in the same nest (WT mice) (Additional file 1: Fig. S1A), micro-CT showed that Tg-hRANKL mice displayed reduced bone mass, destroyed bone microstructure and osteoporotic bone parameters (Fig. 2A–C, F–K). Compared with WT mice, Tg-hRANKL mice had less bone mass in the tibiae, which was displayed in a micro-CT scanner (Fig. 2A and Additional file 1: Fig. S1B). Furthermore, the micro-CT results also showed the destruction of the bone microstructure in Tg-hRANKL mice (Fig. 2B and Additional file 1: Fig. S1B). In addition, compared with WT mice, Tg-hRANKL mice displayed increased Bone Mineral Density (BMD), Bone Volume/Tissue Volume (BV/TV), Trabecular Thickness (Tb.Th), and Trabecular Number (Tb.N) as well as decreased Bone Surface/Bone Volume (BS/BV) and Trabecular Separation (Tb.Sp) (Fig. 2F–K). Moreover, H&E staining also showed the phenotype of bone loss in Tg-hRANKL mice, such as the thinning of the growth plate, thinning and disorder of bone trabeculae, fewer bone trabeculae, enlargement of the bone marrow cavity and an increase in fat (Fig. 2B). Quantitative results showed that Tg-hRANKL mice had a reduced trabecular area (Fig. 2L). More importantly, TRAP staining showed that Tg-hRANKL mice had more osteoclasts than WT mice (Fig. 2C, M). The above results showed that our experimental system is reliable.

Subsequently, using double IF staining, we observed the expression of BCL2 phosphorylated at two sites in the OCPs (RANK was used as the marker) of trabecular bone in Tg-hRANKL mice (shown as overlaps of phosphorylated BCL2 and RANK). Compared with WT mice, Tg-hRANKL mice had more obvious overlapping of phosphorylated BCL2 at S70 and RANK (Fig. 2D and Additional file 1: Fig. S2A). However, compared with WT mice, Tg-hRANKL mice did not show more overlapping fluorescence of phosphorylated BCL2 at S87 and RANK (Fig. 2D and Additional file 1: Fig. S2A). These results indicate that BCL2 at S70, not S87, could be phosphorylated in the OCPs of transgenic mice overexpressing RANKL. After enriching RANK^+^ CSF1R^+^ bone marrow cells (regarded as OCPs in late differentiation), it was found that the alteration of phosphorylated BCL2 at different sites in vitro was consistent with that of phosphorylated BCL2 at different sites in vivo (Fig. 2E, N and Additional file 1: Fig. S2B), confirming the above observation.

### BCL2 mutation at S70, but not S87, inhibited RANKL-induced osteoclastogenesis

**Fig. 3:**
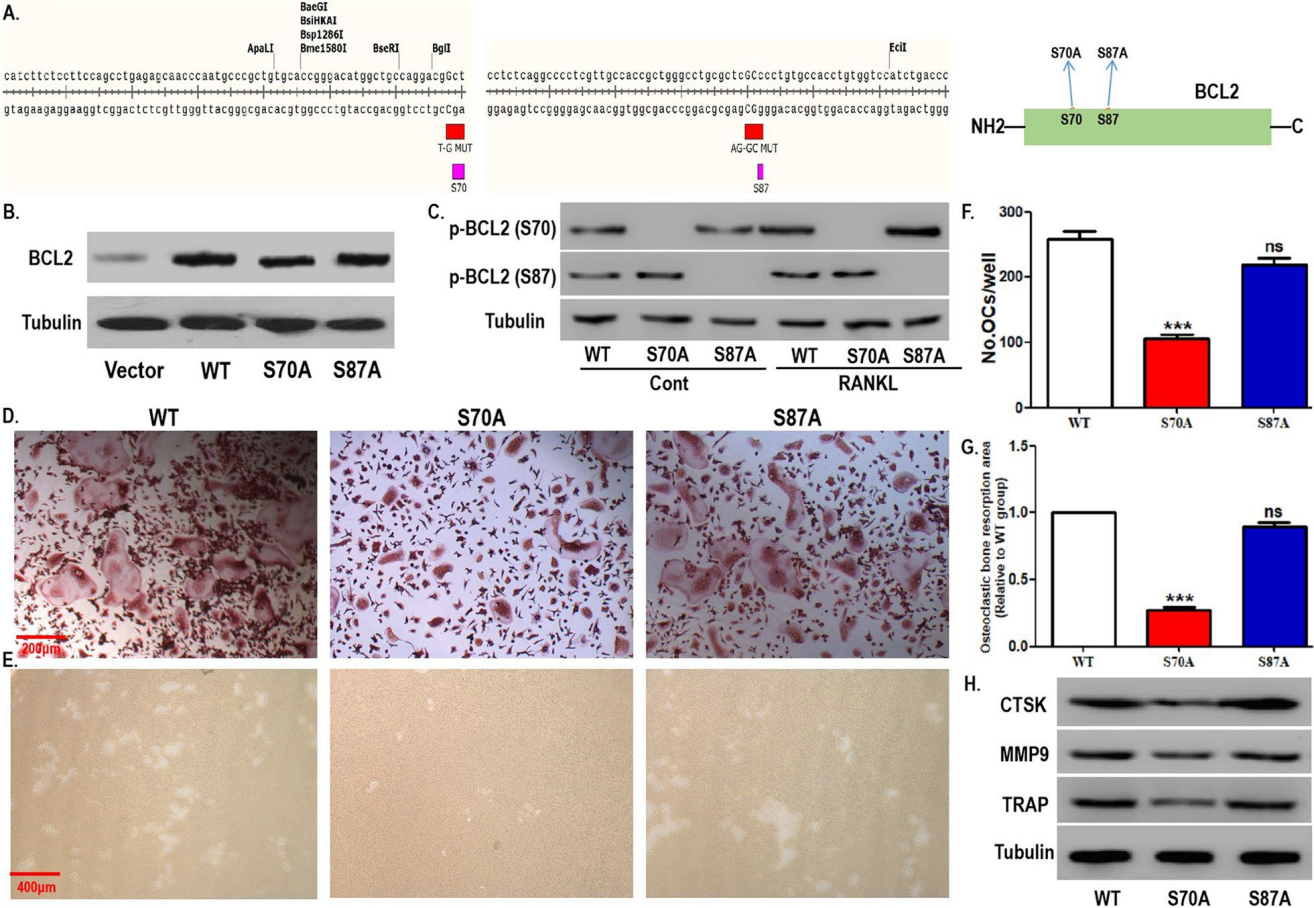
BCL2 mutation at S70, not S87, inhibited RANKL-induced osteoclastogenesis. **A** The working model diagram of site-directed mutagenesis: mutation from S70 to S70A (T-G MUT) or mutation from S87 to S87A (AG-GC MUT). **B** BCL2 expression in OCPs stably transfected with blank vector, wild-type (WT) BCL2, and two mutant BCL2 constructs (S70A or S87A) was detected using western blotting assays. **C** After OCPs transfected with WT BCL2 and the two mutant BCL2 constructs were treated with or without RANKL for 20 min in α-MEM with 1% FBS, p-BCL2 (S70), and p-BCL2 (S87) were detected using western blotting assays. **D** The transfected OCPs were treated with M-CSF plus RANKL for 4 days in α-MEM with 5% FBS. Representative images of TRAP-positive multinucleated cells in each group. Scale bar, 200 μm. **E** The osteoclastic bone resorption activity caused by OCPs inoculated on bone discs and treated with M-CSF plus RANKL for 6 days in α-MEM with 5% FBS was evaluated by scanning electron microscopy. Scale bar, 400 μm. **F** Quantitative results showing the number of TRAP-positive multinucleated cells in **D**. **G** Quantitative results showing the mean resorption pit area in **E**. The resorption pit area was normalized to that of WT OCPs. **H** After the transfected OCPs were treated as described in **D**, the protein expression of CTSK, MMP9, and TRAP was detected using western blotting assays. The experiments were replicated at least three times. Data are presented as the mean ± SEM from three independent experiments. ***P < 0.001. *ns* not significant, *Cont* the control group without RANKL intervention

Using site-directed mutagenesis, we observed the effect of mutations at different phosphorylation sites of BCL2 in OCPs on RANKL-induced osteoclastogenesis. The working model diagram of site-directed mutagenesis is shown in Fig. 3A. First, the overexpression efficiency of OCPs transfected with WT or mutant BCL2 was verified via western blotting assays (Fig. 3B). As shown in Fig. 3C, in the absence of RANKL, OCPs with a BCL2 mutation at S70 did not express phosphorylated BCL2 at S70, while OCPs with a BCL2 mutation at S87 did not express phosphorylated BCL2 at S87. Under RANKL intervention, although the expression level of phosphorylated BCL2 at S70 was significantly increased in WT cells and the S87 site mutant cells, the S70 site mutant cells still lacked the expression of phosphorylated BCL2 at S70 (Fig. 3C). However, RANKL administration did not affect the expression level of phosphorylated BCL2 at S87 in WT and the two types of mutant cells. Accordingly, the mutation efficiency of the two types of mutant cells was identified.

As shown in Fig. 3D, F, under the induction of RANKL plus M-CSF, the number of mature osteoclasts derived from OCPs with BCL2 mutation at S70 decreased significantly, whereas BCL2 mutation at S87 had no effect on the differentiation of OCPs into osteoclasts. Bone resorption assays also showed that OCPs with BCL2 mutation at S70 had smaller resorption pits, while BCL2 mutation at S87 did not affect the areas of resorption pits (Fig. 3E, G). Western blotting assays showed that the protein expression of osteoclastic markers (CTSK, MMP9 and TRAP) in OCPs was significantly inhibited by BCL2 mutation at S70 (Fig. 3H). However, the BCL2 mutation at S87 had no effect on the protein expression of these markers in OCPs (Fig. 3H). These results support the positive effect of BCL2 phosphorylation at S70 on osteoclastogenesis.

### BCL2 mutation at S70 promoted OCP apoptosis while BCL2 mutation at S87 was contrary

**Fig. 4:**
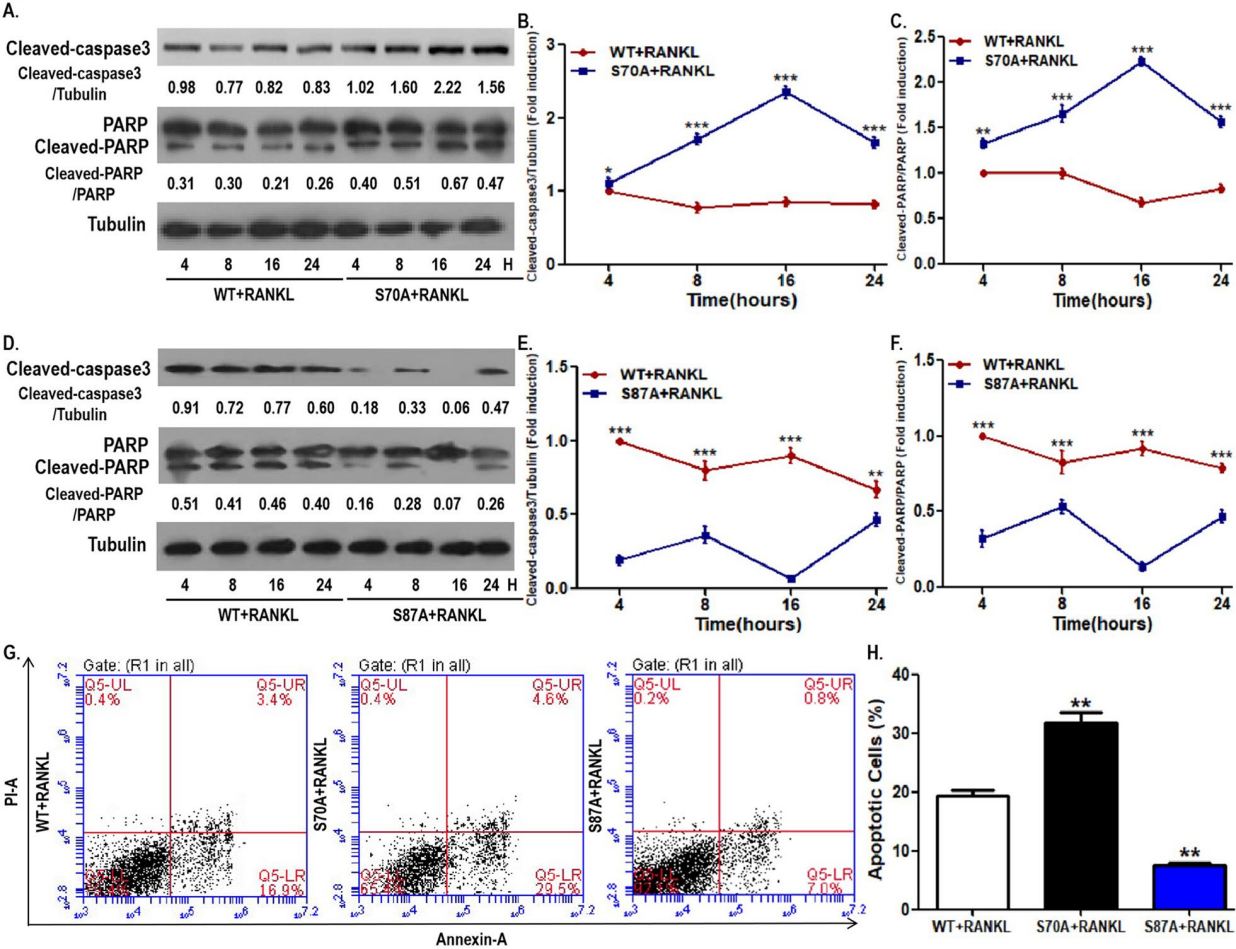
BCL2 mutation at S70 promoted OCP apoptosis while BCL2 mutation at S87 was contrary. **A** OCPs transfected with WT BCL2 or S70A BCL2 were treated with RANKL for the indicated times in α-MEM with 2.5% FBS. Cleaved caspase3 and cleaved PARP were detected using western blotting assays. **B**, **C** Dynamic changes in cleaved caspase3 and cleaved PARP in OCPs between the WT group and the S70A group. The protein expression level was normalized to that of the control samples (4 h in the WT group). **D** OCPs transfected with WT BCL2 or S87A BCL2 were treated with RANKL for the indicated times in α-MEM with 2.5% FBS. Cleaved caspase3 and cleaved PARP were detected using western blotting assays. **E**, **F** Dynamic changes in cleaved caspase3 and cleaved PARP in OCPs between the WT group and the S87A group. The protein expression level was normalized to that of the control samples (4 h in the WT group). **B**, **C**, **E**, **F** At different time points, pairwise comparisons were made between the WT group and the mutant group. **G** The transfected OCPs were treated with RANKL for 48 h in α-MEM with 2.5% FBS. Cell apoptosis was examined by flow cytometry of Annexin/PI staining. **H** The percentages of apoptotic cells (ANNEXIN-positive cells) are shown in the histograms according to the results in **G**. The experiments were replicated at least three times. Data are presented as the mean ± SEM from three independent experiments. ***P < 0.001, **P < 0.01, *P < 0.05

We have identified the inhibitory effect of BCL2 mutation at S70 in OCPs on osteoclastogenic parameters. The intrinsic mechanism underlying the above phenomena requires further investigation. Previous studies showed the antiapoptotic effect of phosphorylated BCL2 at S70 and the proapoptotic effect of phosphorylated BCL2 at S87, respectively [30–32]. Accordingly, we first elucidated the effects of BCL2 mutation at different sites on osteoclastogenesis from the perspective of programmed death. As shown in Fig. 4A–C, compared with the control group, the time variation curves of cleaved PARP and cleaved caspase3 in the S70-mutation group were significantly higher. In contrast, the time curves of cleaved PARP and cleaved caspase3 in the S87-mutant group were significantly lower than those of the control OCPs (Fig. 4D, E). In addition, the AV/PI staining analyses showed that OCPs with BCL2 mutation at S70 had more apoptotic cells, while OCPs with BCL2 mutation at S87 had fewer apoptotic cells (Fig. 4G, H). These results suggest that BCL2 phosphorylation at S70 inhibits OCP apoptosis and BCL2 phosphorylation at S87 promotes OCP apoptosis. In addition, we further evaluated the effect of BCL2 mutation at corresponding sites on the mitochondrial apoptosis of OCPs by detecting the mitochondrial membrane potential. JC-10-related flow cytometry showed that the mitochondrial membrane potential in OCPs with BCL2 mutation at S70 was significantly weaker than that of the control cells (Fig. 6D, E). Nevertheless, the mitochondrial membrane potential in OCPs with BCL2 mutation at S87 was significantly greater than that of the control cells (Fig. 6D, E).

### BCL2 mutation at S70, but not S87, inhibited the autophagic activity of OCPs

**Fig. 5:**
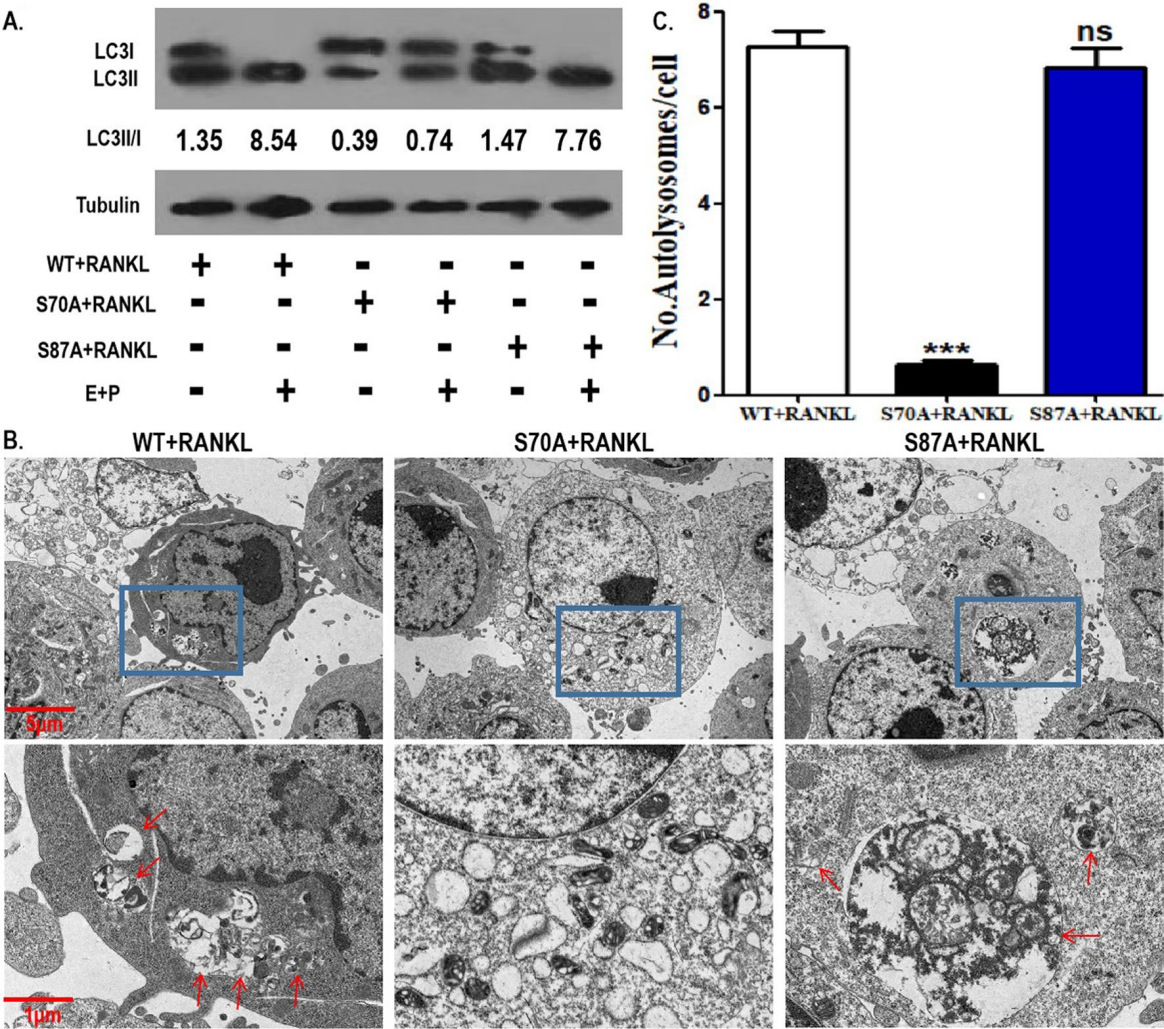
BCL2 mutation at S70, not S87, inhibited the autophagic activity of OCPs. **A** OCPs transfected with WT BCL2 and the two mutant BCL2 were treated with RANKL for 8 h together with or without lysosomal protease inhibitor (E64d plus pepstatin A) in α-MEM with 2.5% FBS. LC3 protein level was detected using western blotting assays. LC3 conversion rate is expressed as the ratio of LC3II to LC3I. **B** The transfected OCPs were treated with RANKL for 24 h in α-MEM with 2.5% FBS. The autolysosomes (red arrows) in OCPs were observed under TEM. Scale bar, 5 μm or 1 μm. **C** The histograms showing quantitative results of autolysosomes in **B** (75 cells from 3 independent experiments). The experiments were replicated at least three times. Data are presented as the mean ± SEM from three independent experiments. ***P < 0.001. *ns* not significant, *E* E64d, *P* pepstatin A

Our team has previously shown that BCL2 phosphorylation-dependent OCP autophagy exists in RANKL-induced osteoclastogenesis (Ke1 et al. 2019). We documented that the BCL2 mutation at S70 inhibits osteoclastogenesis and promotes OCP apoptosis under RANKL intervention. Therefore, the effect of the BCL2 mutation at specific sites on RANKL-induced OCP autophagy also needs to be further clarified. As shown in Fig. 5A, the BCL2 mutation at S70 suppressed the LC3 conversion rate (shown as LC3II/I) in OCPs in the absence or presence of lysosomal protease inhibitor (E64d plus pepstatin A) (Fig. 5A). However, the BCL2 mutation at S87 did not affect LC3 conversion of OCPs in the absence or presence of E64d plus pepstatin A (Fig. 5A). Consistently, the BCL2 mutation at S70 suppressed the formation of autolysosomes in OCPs, while the BCL2 mutation at S87 had no effect on autolysosome formation (Fig. 5B, C). E64d plus pepstatin A, increased the ratio of LC3II/I under three experimental conditions (control, S70 mutation and S87 mutation), which showed that autophagic flux was smooth in our experimental system (Fig. 5A). Therefore, the above results suggest that BCL2 phosphorylation at S70 mediates RANKL-induced OCP autophagy.

### BCL2 mutation at S70 enhanced the coimmunoprecipitation of BCL2 and Beclin1 in OCPs

**Fig. 6:**
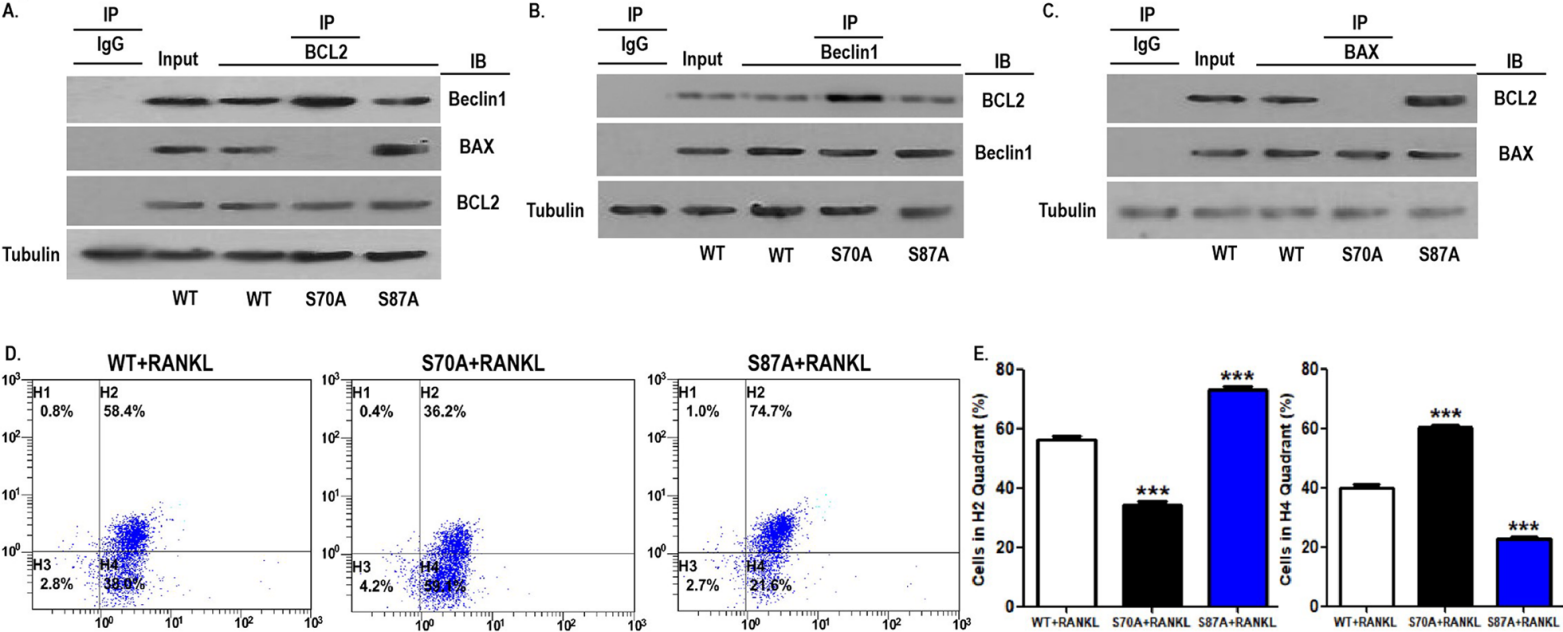
BCL2 mutation at S70 enhanced the coimmunoprecipitation of BCL2 and Beclin1 in OCPs. **A**–**C** The transfected OCPs were treated with RANKL for 2 h in α-MEM with 1% FBS. Cell lysates were extracted for Co-IP with anti-BCL2, anti-Beclin1 or anti-BAX antibody, and subsequently, precipitates were observed using western blotting assays with anti-Beclin1, anti-BAX or anti-BCL2 antibody, respectively. **D** The transfected OCPs were treated with RANKL for 24 h in α-MEM with 2.5% FBS. The mitochondrial membrane potential was detected by measuring the ratio of red/green fluorescence intensity using JC-10 kit, and quantitatively analysed using flow cytometry. **E** The percentages of cells in each quadrant are shown in the histograms according to the results in **D**. The larger proportion of cells in H2 quadrant indicates that mitochondrial membrane potential is higher; The larger proportion of cells in H4 quadrant indicates that mitochondrial membrane potential is lower. The experiments were replicated at least three times. Data are presented as the mean ± SEM from three independent experiments. ***P < 0.001. *IP* the antibody for immunoprecipitation, *IB* the antibody for immunoblot

The positive role of BCL2 phosphorylation at S70 in RANKL-induced OCP autophagy and the promoting role of BCL2 phosphorylation at S87 in OCP apoptosis were confirmed under RANKL intervention. Previous studies have clarified that Beclin1 and BAX were dissociated following BCL2 phosphorylation, which is involved in autophagy activation and apoptotic signal transduction, respectively (Bassik et al. 2004; Wei et al. 2008a, b; Levine et al. 2008). Thus, we observed the effects of the BCL2 mutations at different sites on the BCL2-Beclin1 complex and BCL2-BAX complex in OCPs via coimmunoprecipitation assays. As shown in Fig. 6A, the BCL2 mutation at S70 enhanced the coimmunoprecipitation ability of the BCL2 protein and Beclin1 protein, while the BCL2 mutation at S87 did not affect the coimmunoprecipitation of BCL2 and Beclin1 in OCPs. Nevertheless, the BCL2 mutation at S87 promoted the coimmunoprecipitation of BCL2 and BAX, while the BCL2 mutation at S70 attenuated the coimmunoprecipitation of BCL2 and BAX (Fig. 6A). Using an immunoprecipitation (IP) antibody against Beclin1, the promotion of the BCL2 mutation at S70 on the coimmunoprecipitation of Beclin1 and BCL2 and the ineffectiveness of the BCL2 mutation at S87 on the coimmunoprecipitation of the two molecules were also observed in OCPs (Fig. 6B). Similarly, using an IP antibody against BAX, it was observed that the BCL2 mutation at S87 enhanced the coimmunoprecipitation of BAX and BCL2 and that the BCL2 mutation at S70 reduced their coimmunoprecipitation level in OCPs (Fig. 6C). These results support that BCL2 phosphorylation at S70 can dissociate the BCL2-Beclin1 complex and BCL2 phosphorylation at S87 can dissociate the BCL2-BAX complex.

### OCP autophagy and osteoclastogenesis inhibited by BCL2 mutation at S70 were reversed by TAT-Beclin1

**Fig. 7:**
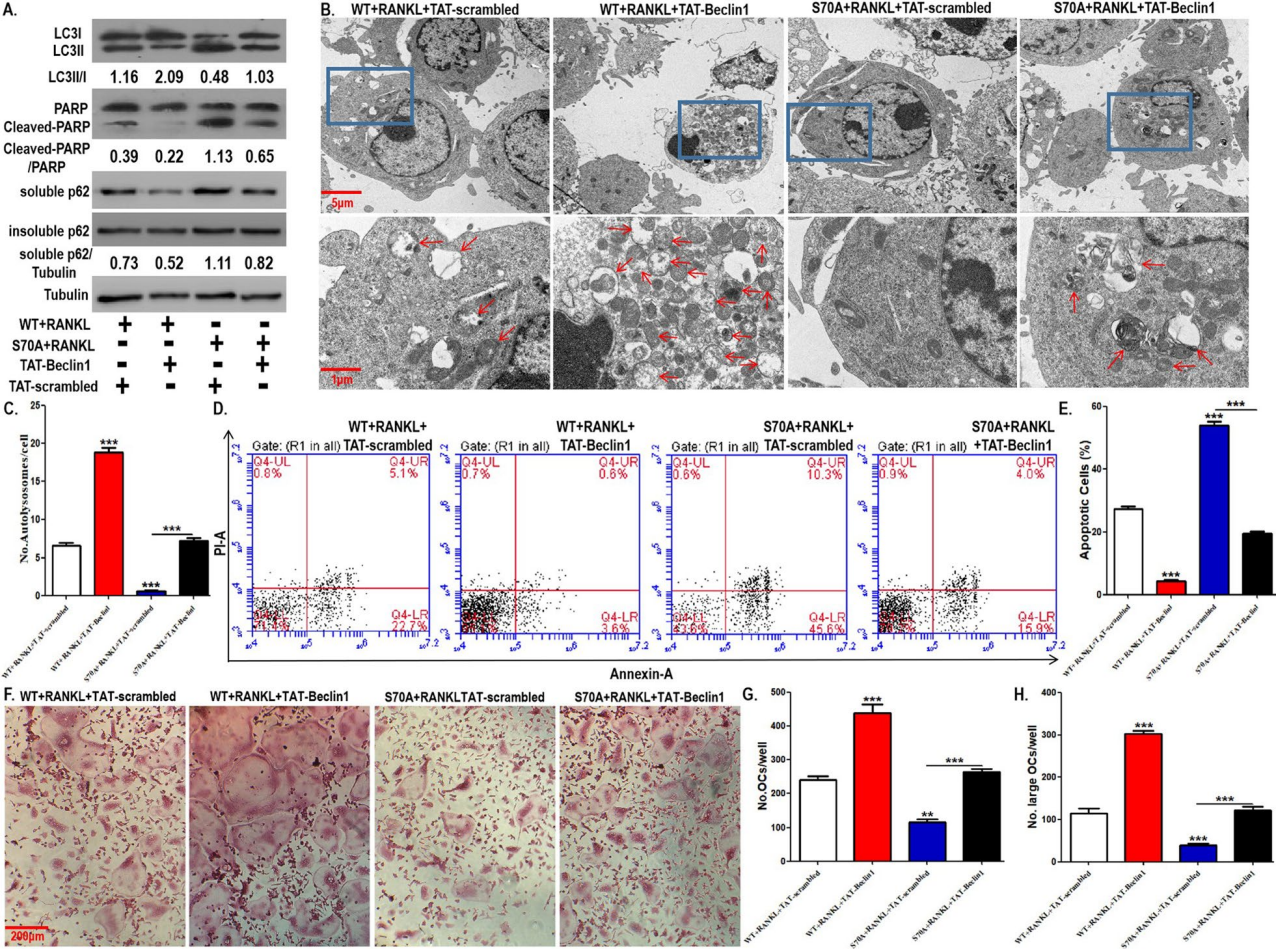
OCP autophagy and osteoclastogenesis inhibited by BCL2 mutation at S70 was reversed by TAT-Beclin1. **A** OCPs transfected with WT BCL2 or S70A BCL2 were treated with RANKL along with TAT-Beclin1 (10 μM) or the control peptide (TAT-scrambled) for 8 h in α-MEM with 2.5% FBS. LC3, cleaved PARP, and p62 (soluble and insoluble p62) were detected using western blotting assays. LC3 conversion rate is expressed as the ratio of LC3II to LC3I. **B** The transfected OCPs were treated with RANKL along with TAT-Beclin1 or TAT-scrambled for 24 h in α-MEM with 2.5% FBS. The autolysosomes (red arrows) in OCPs were observed under TEM. Scale bar, 5 μm or 1 μm. **C** The histograms showing quantitative results of autolysosomes in **B** (75 cells from 3 independent experiments). **D** The transfected OCPs were treated with RANKL along with TAT-Beclin1 or TAT-scrambled for 48 h in α-MEM with 2.5% FBS. Cell apoptosis was examined by flow cytometry of Annexin/PI staining. **E** The percentages of apoptotic cells (ANNEXIN-positive cells) are shown in the histograms according to the results in **D**. **F** The transfected OCPs were treated with M-CSF plus RANKL along with TAT-Beclin1 or TAT-scrambled for 4 days in α-MEM with 5% FBS. Representative images of TRAP-positive multinucleated cells in each group. Scale bar, 200 μm. **G**, **H** Quantitative results regarding mature osteoclasts (more than 3 nuclei) or large osteoclasts (more than 5 nuclei) in **F**. The experiments were replicated at least three times. Data are presented as the mean ± SEM from three independent experiments. ***P < 0.001, **P < 0.01

The upregulation of BCL2 phosphorylation at S70 on RANKL-induced OCP autophagy and osteoclastogenesis has been clarified. Moreover, BCL2 phosphorylation at S70 led to the release of the Beclin1 molecule from the BCL2-Beclin1 complex. We combined BCL2 mutation at S70 and pharmacological overexpression of Beclin1 to further confirm the role of autophagy in osteoclastogenesis regulated by BCL2 mutation at S70. TAT-Beclin1, as a Beclin1 recombinant protein, can activate autophagy (Shoji-Kawata et al. 2013; Sun et al. 2018; Atwood et al. 2020). Here, TAT-Beclin1 was applied as a tool to overexpress Beclin1 in OCPs. As shown in Fig. 7A, BCL2 mutation at S70 inhibited LC3 transformation and autolysosome formation, which was reversed by TAT-Beclin1 administration to OCPs (Fig. 7A–C). Furthermore, the protein expression of soluble p62 promoted by BCL2 mutation at S70 was recovered by TAT-Beclin1 administration, which demonstrated the stability of autophagic flux (Fig. 7A). The protein expression of insoluble p62 remained stable under various treatments (Fig. 7A). The above results confirmed the reliability of our experimental system. The TRAP staining results showed that BCL2 mutation at S70 obviously reduced the number of differentiated osteoclasts and large osteoclasts, which was reversed by TAT-Beclin1 administration (Fig. 7F–H). Bone resorption experiments also showed that BCL2 mutation at S70 significantly inhibited the formation of bone resorption pits, which were also recovered by TAT-Beclin1 administration (Additional file 1: Fig. S3A, B). In addition, in OCPs, the expression level of cleaved PARP upregulated by BCL2 mutation at S70 was reversed by TAT-Beclin1 administration (Fig. 7A). The AV/PI staining results also showed that the apoptotic OCPs increased by BCL2 mutation at S70 were recovered by TAT-Beclin1 administration (Fig. 7D, E). Together, these results further suggest that BCL2 phosphorylation at S70 can promote the release of Beclin1 from the BCL2-Beclin1 complex and can subsequently activate OCP autophagy, which contributes to apoptotic resistance and osteoclastogenesis.

## Discussion

RANKL can promote the autophagy of OCPs through BCL2 phosphorylation, which exists in RANKL-induced osteoclastogenesis (Ke et al. 2019a). However, the significance of BCL2 for osteoclastogenesis includes both positive and negative effects, which means that the inhibition of BCL2 is not the best choice for the treatment of osteoclastic bone loss (Bozec et al. 2008; Yamashita et al. 2008). BCL2 phosphorylation is known to promote the dissociation of Beclin1 and proapoptotic molecules (Bassik et al. 2004; Wei et al. 2008a, b; Levine et al. 2008). Moreover, the cell survival direction caused by BCL2 phosphorylation at different sites is also different: BCL2 phosphorylated at the S70 site is antiapoptotic, while BCL2 phosphorylated at the S87 site promotes apoptosis (Liu et al. 2010; Liu et al. 2018; Saatci et al. 2018). The above phenomena led to an interesting scientific question for our study: Is the BCL2 phosphorylation level at different sites discrepant after RANKL intervention in OCPs? In addition, under RANKL intervention, whether phosphorylated BCL2 at a specific site is conducive to osteoclastogenesis is also worthy of further exploration. This study focused on the application of Tg-hRANKL mice and the S70 or S87 site-directed mutant OCPs to clarify the underlying mechanism of BCL2-related osteoclastogenesis at the molecular, cellular and animal levels for the first time.

First, RANKL promoted BCL2 phosphorylation at the S70 site in OCPs in vitro and in vivo. However, RANKL did not affect BCL2 phosphorylation at S87. These results are similar to the previous study. Our team previously demonstrated on RAW264.7-derived OCPs that RANKL can promote BCL2 phosphorylation at the S70 site in a time-dependent and concentration-dependent manner (Ke et al. 2019a). Considering that BCL2 phosphorylation at S70 plays an antiapoptotic role, the promoting effect of RANKL on osteoclastogenesis through BCL2 signalling may be based on BCL2 phosphorylation at the S70 site. As expected, our experimental data confirmed that the mutation of BCL2 at S70 inhibited RANKL-induced osteoclastogenesis and bone resorption activity. Furthermore, BCL2 at the S87 site, which was not influenced by RANKL, did not affect osteoclastogenic parameters following its mutation. In addition, BCL2 mutation at S70 significantly promoted the apoptotic parameters of OCPs. In contrast, the BCL2 mutation at S87 showed the opposite effect on OCP apoptosis. These results are consistent with other studies. Piperlongumine inhibits apoptosis by phosphorylating BCL2 at S70 in a mouse model of rotenone-induced Parkinson’s disease (Liu et al. 2018). Moreover, BCL2 phosphorylation at S70 directly regulates apoptosis by disrupting the binding to the proapoptotic protein in HER2-positive breast cancer cells (Saatci et al. 2018). In contrast, the trend of cleaved PARP was directly proportional to that of the BCL2 phosphorylation level at S87 in HEK293 cells treated with Tau or PP2Ac (Liu et al. 2010). Our data identified a similar effect of BCL2 phosphorylation at the S70 or S87 site on apoptosis regulation in the field of osteoclastogenesis. The above results indicate that BCL2 phosphorylation at S70 contributes to osteoclastogenesis, on which the apoptosis resistance of BCL2 phosphorylation at S70 exerts a key effect. Remarkably, BCL2 mutation at S87 inhibited OCP apoptosis but had no effect on osteoclastogenic capacity. We consider that this is because RANKL does not affect BCL2 phosphorylation at S87 in OCPs, which causes the BCL2 mutation at S87 to affect OCP apoptosis while failing to effectively affect the whole process of osteoclastogenesis.

Protective autophagy can act as an antiapoptotic mechanism. Autophagy can protect cancer cells from apoptosis, which has been proven (Fitzwalter and Thorburn 2015). In cancer treatment, autophagy inhibition mediates apoptosis sensitization by reducing the conversion of FOXO3a (Fitzwalter et al. 2018). As an effective therapy vector, autophagy-inhibitory polymer have inherent apoptosis-sensitization capacity (Wang et al. 2020). In addition, autophagy protects tumours from T cell-mediated cytotoxicity by inhibiting TNFα-induced apoptosis (Young et al. 2020). Importantly, under RANKL induction, not only do autophagy activation and apoptosis inhibition coexist, but also the autophagy activation can play an antiapoptotic role in OCPs (Ke et al. 2019a; b). BCL2 phosphorylation at S70 is known to play a role in antiapoptotic function in OCPs. Does BCL2 phosphorylation at S70 mediate RANKL-induced OCP autophagy? Our results showed that BCL2 mutation at S70 inhibited the autophagy activity of OCPs, while BCL2 mutation at S87 did not affect OCP autophagy. Thus, the upregulation of BCL2 phosphorylation at S70 on OCP autophagy was also confirmed. Moreover, the promoting effect of BCL2 mutation at S70 on the apoptotic parameters of OCPs was reversed by autophagy activation with TAT-Beclin1, which indicates that RANKL could promote autophagy through BCL2 phosphorylation at S70, thereby inhibiting apoptosis in OCPs. Importantly, the inhibitory effect of BCL2 mutation at S70 on osteoclastogenic parameters was reversed by TAT-Beclin1 administration, which further indicates that RANKL promoted osteoclastogenesis through BCL2 phosphorylation at S70-autophagy activation signal transduction.

**Fig. 8:**
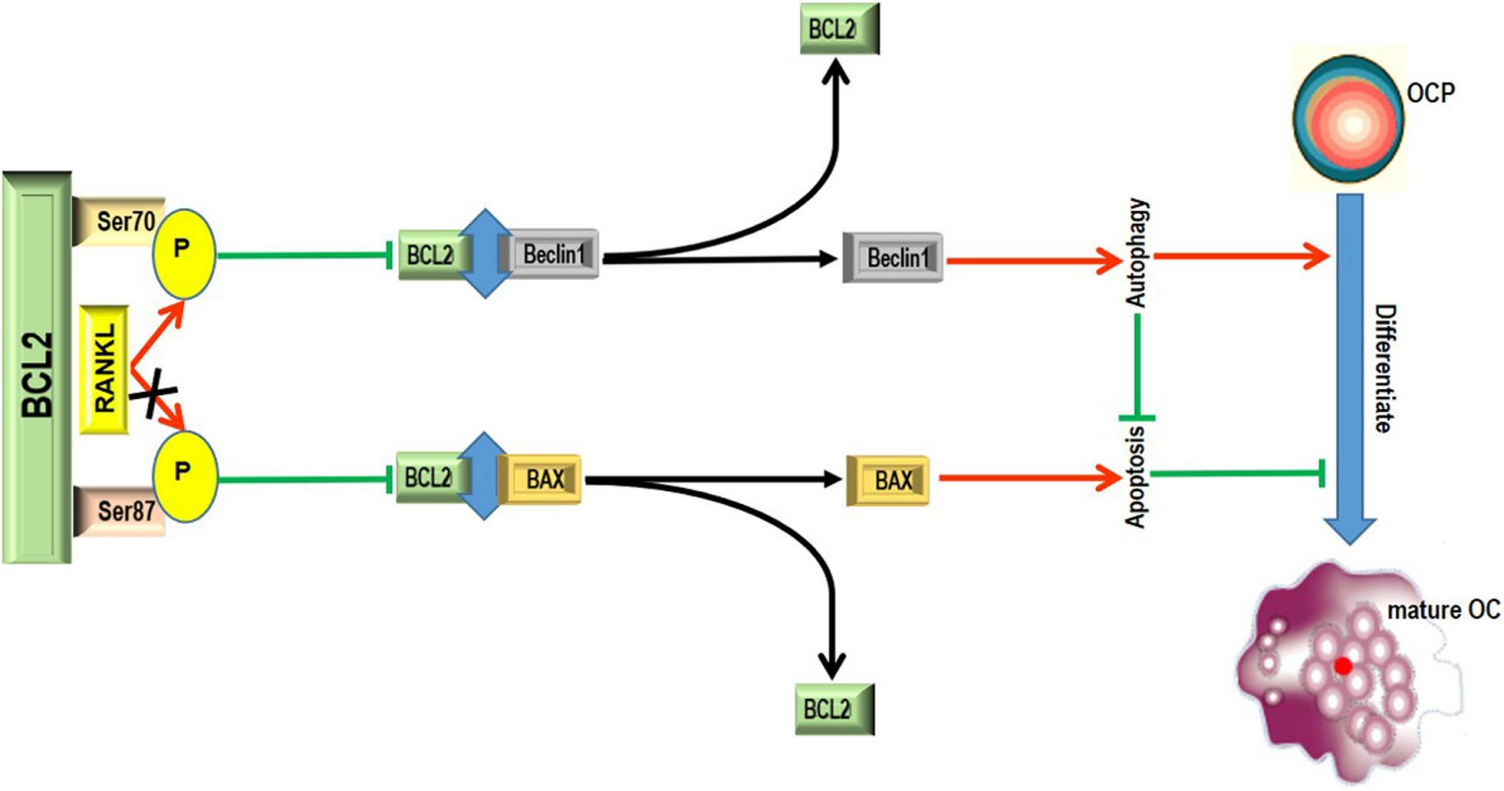
The schematic diagram representing the current working model in our study. In brief, BCL2 phosphorylation at Ser87 can dissociate the BCL2-BAX complex, subsequently making proapoptotic molecule BAX lead to apoptotic signal transduction and promote apoptosis, which is not conducive to the differentiation of OCPs into mature osteoclasts. BCL2 phosphorylation at Ser70 can dissociate the BCL2-Beclin1 complex, enabling autophagic molecule Beclin1 to enter autophagic flux and activate autophagy, which represses apoptosis and is conducive to osteoclastic differentiation. RANKL is a stimulator of BCL2 phosphorylation at Ser70, but not Ser87. Therefore, RANKL can induce osteoclastogenesis by phosphorylating BCL2 at Ser70. *P* phosphorylation, *OCP* osteoclast precursor, *OC* osteoclast

BCL2 phosphorylation is known to promote the dissociation of autophagic molecule Beclin1 from the BCL2-Beclin1 complex, resulting in the entry of Beclin1 into autophagic flux and subsequent autophagy activation (Pattingre et al. 2005; Wei et al. 2008a, b; Liu et al. 2013; Ke et al. 2019b). As an antiapoptotic molecule, BCL2 can prevent subsequent apoptotic signal transduction by binding to proapoptotic proteins such as BAX (Bassik et al. 2004; Wei et al. 2008b). However, BCL2 phosphorylation has the ability to promote the double dissociation of the BCL2-Beclin1 complex and BCL2-BAX complexes, which can regulate both survival and apoptosis (Wei et al. 2008b; Levine et al. 2008). In this study, it was elaborated that BCL2 mutation at S70 increased the coimmunoprecipitation level of BCL2 and Beclin1 in OCPs. However, the BCL2 mutation at S87 increased the coimmunoprecipitation level of BCL2 and BAX in OCPs. These results explain the regulatory effects of BCL2 mutation at different sites on autophagy and apoptosis in OCPs. It could be inferred that BCL2 phosphorylation at S70 inhibits the interaction between BCL2 and Beclin1, resulting in Beclin1-dependent autophagy activation in OCPs. In contrast, BCL2 phosphorylation at S87 inhibited the interaction between BCL2 and BAX in OCPs, resulting in increased apoptosis. Remarkably, the BCL2 mutation at S87 significantly increased the mitochondrial membrane potential, while the BCL2 mutation at S70 showed the opposite effect in OCPs. Proapoptotic proteins are required for mitochondrial membrane permeabilization after different apoptosis induction signals (Wei et al. 2001; Levine et al. 2008). Based on the coupling effect, antiapoptotic proteins in other cellular compartments could hinder the movement of proapoptotic proteins to mitochondria (Levine et al. 2008). Accordingly, BCL2 phosphorylation at S87 caused a decline in mitochondrial membrane potential in OCPs, resulting from the dissociation of BAX from the BCL2-BAX complex, which ultimately led to mitochondrial apoptosis. Given the inhibitory effect of BCL2 mutation at S70 on the mitochondrial membrane potential in OCPs, BCL2 phosphorylation at S70 could enhance the mitochondrial membrane potential in OCPs. Furthermore, the coimmunoprecipitation ability between BCL2 and BAX was inhibited by BCL2 mutation at S70 in OCPs. Therefore, we consider that this may be because BCL2 phosphorylation at S70 promotes the dissociation of the BCL2-Beclin1 complex, enabling more BCL2 molecules to participate in the binding with proapoptotic proteins and finally inhibiting mitochondrial membrane permeabilization in OCPs. A previous study also demonstrated that the promotion of the interaction between Beclin1 and BCL2 can weaken the interaction between BCL2 and BAX to activate BAX and induce apoptosis (Maejima et al. 2013). Based on the classical viewpoint, our experimental data mechanistically confirm that the regulation of BCL2 phosphorylation at S70 on autophagy activity affects OCP apoptosis. Furthermore, Beclin1 overexpression with TAT-Beclin1 not only reversed the inhibitory effect of BCL2 mutation at S70 on OCP autophagy but also recovered the promotion of OCP apoptosis and the inhibition of osteoclastogenesis by BCL2 mutation at S70. It could be inferred that RANKL promotes Beclin1-dependent protective autophagy of OCPs through BCL2 phosphorylation at S70, thereby enhancing osteoclastogenesis. Overall, our study confirmed that BCL2 phosphorylation at S70 can release Beclin1 and induce autophagic flux in OCPs, subsequently activating autophagy and ultimately repressing apoptosis and promoting osteoclastogenesis. Our current working model regarding the respective roles of BCL2 phosphorylation at Ser70 or Ser87 in osteoclastogenesis is illustrated in Fig. 8.

## Conclusion

In the present study, the role of BCL2 phosphorylation at different sites in osteoclastogenesis and its underlying mechanism are described. According to the overall data, BCL2 phosphorylation at the S70 site mediates RANKL-induced OCP autophagy due to its positive regulation of the BCL2-Beclin1 signalling pathway, thereby resisting the apoptosis of OCPs and promoting osteoclastogenesis. This study is the first to clarify the long-standing doubts about the specific role of the BCL2 molecule and its phosphorylation modification in osteoclastogenesis, which provides more potential clues for the clinical diagnosis and treatment of pathological bone loss dominated by osteoclastic bone resorption.

## Supplementary Information

**Additional file 1: Figure S1.** Tg-hRANKL mice had less bone mass and destructive bone microstructure. **Figure S2.** Supplementary data on BCL2 phosphorylation in Tg-hRANKL mice. **Figure S3.** Bone resorptive activity inhibited by BCL2 mutation at S70 was reversed by TAT-Beclin1.

## Data Availability

The raw data supporting the conclusions of this manuscript will be made available by the authors, without undue reservation, to any qualified researcher.

## Abbreviations

RANKL: Receptor activator of nuclear factor-κB ligand
OCPs: Osteoclast precursors
BCL2: B-cell lymphoma 2
S70: Ser70 site
S87: Ser87 site
WT: Wild-type
CTSK: Cathepsin K
MMP9: Matrix metallopeptidase 9
TRAP: Tartrate resistant acid phosphatase
α-MEM: Alfa-minimum essential medium
FBS: Fetal bovine serum
BMMs: Bone marrow-derived macrophages
PVDF: Polyvinylidene fluoride membranes
HRP: Horseradish peroxidase
TEM: Transmission electron microscopy
Micro-CT: Micro-computed tomography
BMD: Bone mineral density
BV/TV: Bone volume/tissue volume
BS/BV: Bone surface/bone volume
Tb.Th: Trabecular thickness
Tb.N: Trabecular number
Tb.Sp: Trabecular separation
H&E: Haematoxylin and eosin
IF: Immunofluorescence
FACS: Fluorescence-activated cell sorting
BSA: Bovine serum albumin

## References

[CR1] Arai A (2019). Beclin1 modulates bone homeostasis by regulating osteoclast and chondrocyte differentiation. J Bone Miner Res.

[CR2] Atwood DJ (2020). Increased mTOR and suppressed autophagic flux in the heart of a hypomorphic Pkd1 mouse model of autosomal dominant polycystic kidney disease. Cell Signal.

[CR3] Bassik M (2004). Phosphorylation of BCL-2 regulates ER Ca2+ homeostasis and apoptosis. EMBO J.

[CR4] Bonnet N (2019). RANKL inhibition improves muscle strength and insulin sensitivity and restores bone mass. J Clin Invest.

[CR5] Boyle WJ (2003). Osteoclast differentiation and activation. Nature.

[CR6] Bozec A (2008). Osteoclast size is controlled by Fra-2 through LIF/LIF-receptor signalling and hypoxia. Nature.

[CR7] Chawla S (2019). Denosumab in patients with giant-cell tumour of bone: a multicentre, open-label, phase 2 study. Lancet Oncol.

[CR8] Cheng L (2020). Oestrogen-activated autophagy has a negative effect on the anti-osteoclastogenic function of oestrogen. Cell Prolif.

[CR9] Chung YH (2014). Beclin-1 is required for RANKL-induced osteoclast differentiation. J Cell Physiol.

[CR10] Cummings SR (2009). Denosumab for prevention of fractures in postmenopausal women with osteoporosis. N Engl J Med.

[CR11] David JP (2002). JNK1 modulates osteoclastogenesis through both c-Jn phosphorylation-dependent and -independent mechanisms. J Cell Sci.

[CR12] DeSelm CJ (2011). Autophagy proteins regulate the secretory component of osteoclastic bone resorption. Dev Cell.

[CR13] Eghbali-Fatourechi G, Khosla S, Sanyal A (2003). ole of RANK ligand in mediating increased bone resorption in early postmenopausal women. J Clin Invest.

[CR14] Fitzwalter BE, Thorburn A (2015). Recent insights into cell death and autophagy. FEBS J.

[CR15] Fitzwalter BE (2018). Autophagy inhibition mediates apoptosis sensitization in cancer therapy by relieving FOXO3a turnover. Dev Cell.

[CR16] Fujii T (2021). MEF2C regulates osteoclastogenesis and pathologic bone resorption via c-FOS. Bone Res.

[CR17] Ha J (2010). CXC chemokine ligand 2 induced by receptor activator of NF-kappa B ligand enhances osteoclastogenesis. J Immunol.

[CR18] Ikeda F (2004). Critical roles of c-Jun signaling in regulation of NFAT family and RANKL-regulated osteoclast differentiation. J Clin Invest.

[CR19] Ke D (2019). JNK1 regulates RANKL-induced osteoclastogenesis via activation of a novel Bcl-2-Beclin1-autophagy pathway. FASEB J.

[CR20] Ke D (2019). Autophagy mediated by JNK1 resists apoptosis through TRAF3 degradation in osteoclastogenesis. Biochimie.

[CR21] Levine B, Sinha SC, Kroemer G (2008). Bcl-2 family members: dual regulators of apoptosis and autophagy. Autophagy.

[CR22] Lin NY (2016). Inactivation of autophagy ameliorates glucocorticoid-induced and ovariectomy-induced bone loss. Ann Rheum Dis.

[CR23] Liu XA (2010). Tau dephosphorylation potentiates apoptosis by mechanisms involving a failed dephosphorylation/activation of Bcl-2. J Alzheimers Dis.

[CR24] Liu H (2013). Interleukin 17A inhibits autophagy through activation of PIK3CA to interrupt the GSK3B-mediated degradation of BCL2 in lung epithelial cells. Autophagy.

[CR25] Liu J (2018). Piperlongumine restores the balance of autophagy and apoptosis by increasing BCL2 phosphorylation in rotenone-induced Parkinson disease models. Autophagy.

[CR26] Lu X (2009). Fibroblast growth factor receptor 1 regulates the differentiation and activation of osteoclasts through Erk1/2 pathway. Biochem Biophys Res Commun.

[CR27] Maejima Y (2013). Mst1 inhibits autophagy by promoting the interaction between Beclin1 and Bcl-2. Nat Med.

[CR28] Pattingre S (2005). Bcl-2 anti-apoptotic proteins inhibit Beclin1-dependent autophagy. Cell.

[CR29] Raje N (2018). Denosumab versus zoledronic acid in bone disease treatment of newly diagnosed multiple myeloma: an international, double-blind, double-dummy, randomised, controlled, phase 3 study. Lancet Oncol.

[CR30] Raje NS, Bhatta S, Terpos E (2019). Role of the RANK/RANKL pathway in multiple myeloma. Clin Cancer Res.

[CR31] Rinotas V (2014). Novel genetic models of osteoporosis by overexpression of human RANKL in transgenic mice. J Bone Miner Res.

[CR32] Roux S, Mariette X (2010). RANK and RANKL expression in giant-cell tumour of bone. Lancet Oncol.

[CR33] Saatci Ö (2018). Targeting PLK1 overcomes T-DM1 resistance via CDK1-dependent phosphorylation and inactivation of Bcl-2/xL in HER2-positive breast cancer. Oncogene.

[CR34] Shoji-Kawata S (2013). Identification of a candidate therapeutic autophagy-inducing peptide. Nature.

[CR35] Sun Y (2018). Beclin-1-dependent autophagy protects the heart during sepsis. Circulation.

[CR36] Takeuchi T (2019). Effects of the anti-RANKL antibody denosumab on joint structural damage in patients with rheumatoid arthritis treated with conventional synthetic disease-modifying antirheumatic drugs (DESIRABLE study): a randomised, double-blind, placebo-controlled phase 3 trial. Ann Rheum Dis.

[CR37] Van Tuyl LH (2010). Baseline RANKL:OPG ratio and markers of bone and cartilage degradation predict annual radiological progression over 11 years in rheumatoid arthritis. Ann Rheum Dis.

[CR38] Wang J (2020). Autophagy-inhibiting polymer as an effective nonviral cancer gene therapy vector with inherent apoptosis-sensitizing ability. Biomaterials.

[CR39] Wei MC (2001). Proapoptotic BAX and BAK: a requisite gateway to mitochondrial dysfunction and death. Science.

[CR40] Wei Y (2008). JNK1-mediated phosphorylation of Bcl-2 regulates starvation-induced autophagy. Mol Cell.

[CR41] Wei Y (2008). Dual role of JNK1-mediated phosphorylation of Bcl-2 in autophagy and apoptosis regulation. Autophagy.

[CR42] Xiong J (2018). Soluble RANKL contributes to osteoclast formation in adult mice but not ovariectomy-induced bone loss. Nat Commun.

[CR43] Xiu Y (2014). Chloroquine reduces osteoclastogenesis in murine osteoporosis by preventing TRAF3 degradation. J Clin Invest.

[CR44] Yamashita J (2008). Role of Bcl2 in osteoclastogenesis and PTH anabolic actions in bone. J Bone Miner Res.

[CR45] Yang X, Chan C (2009). Repression of PKR mediates palmitate-induced apoptosis in HepG2 cells through regulation of Bcl-2. Cell Res.

[CR46] Yang W (2021). TAZ inhibits osteoclastogenesis by attenuating TAK1/NF-κB signaling. Bone Res.

[CR47] Young TM (2020). Autophagy protects tumors from T cell-mediated cytotoxicity via inhibition of TNFα-induced apoptosis. Sci Immunol..

[CR48] Yu W (2021). Bone marrow adipogenic lineage precursors promote osteoclastogenesis in bone remodeling and pathologic bone loss. J Clin Invest.

