# Supplementary material for: Phosphorylation of BCL2 at the Ser70 site mediates RANKL-induced osteoclast precursor autophagy and osteoclastogenesis
Source: Mol Med. 2022 Feb 19;28:22. doi: 10.1186/s10020-022-00449-w (PMC8858497; doi:10.1186/s10020-022-00449-w)
Supplement: Supplementary file 1 — Additional file 1: Figure S1. Tg-hRANKL mice had less bone mass and destructive bone microstructure. Figure S2. Supplementary data on BCL2 phosphorylation in Tg-hRANKL mice. Figure S3. Bone resorptive activity inhibited by BCL2 mutation at S70 was reversed by TAT-Beclin1. [file 10020_2022_449_MOESM1_ESM.docx]

**Supplementary materials**


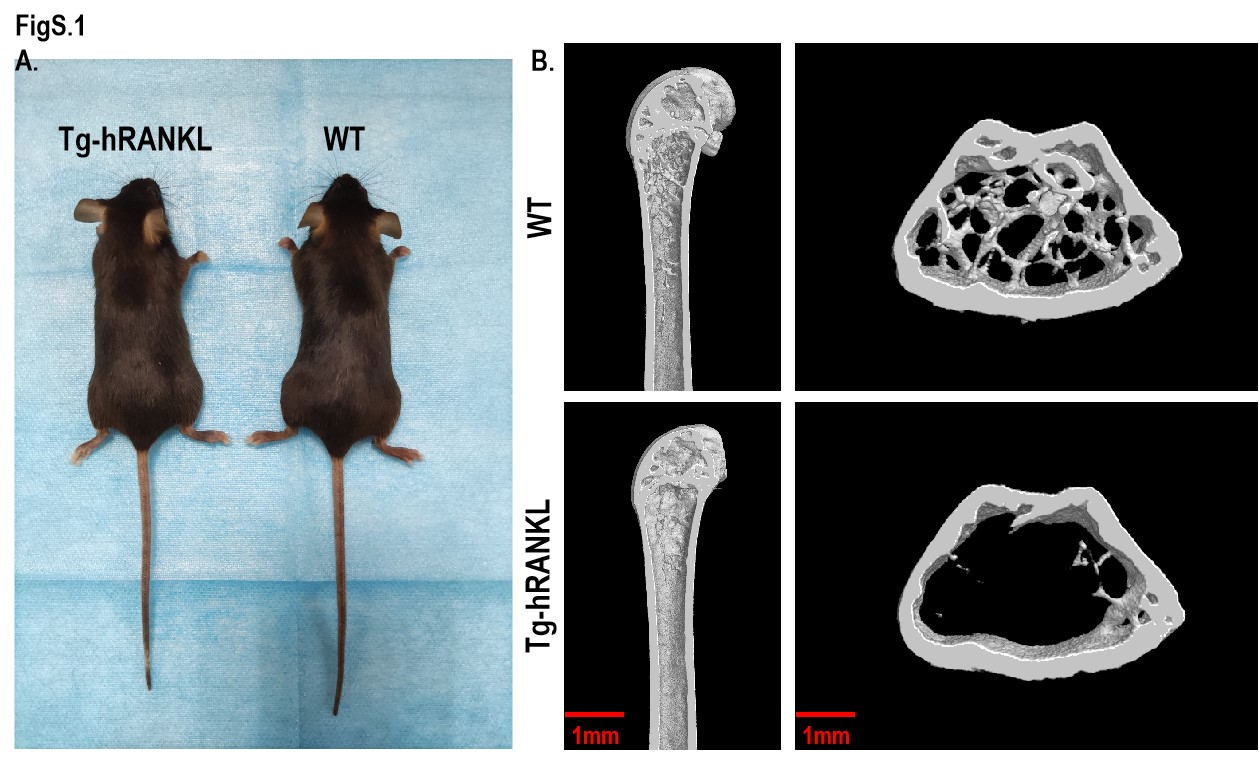


**Fig. S1. Tg-hRANKL mice had less bone mass and destructive bone microstructure.** (A) The appearance of Tg-hRANKL mice and control mice in the same nest (WT mice). (B) Representative 3D Micro-CT reconstructed images of the tibiae from Tg-hRANKL mice and WT mice showing bone mass and bone microstructure (N = 6/group). Scale bar, 2mm or 1mm.


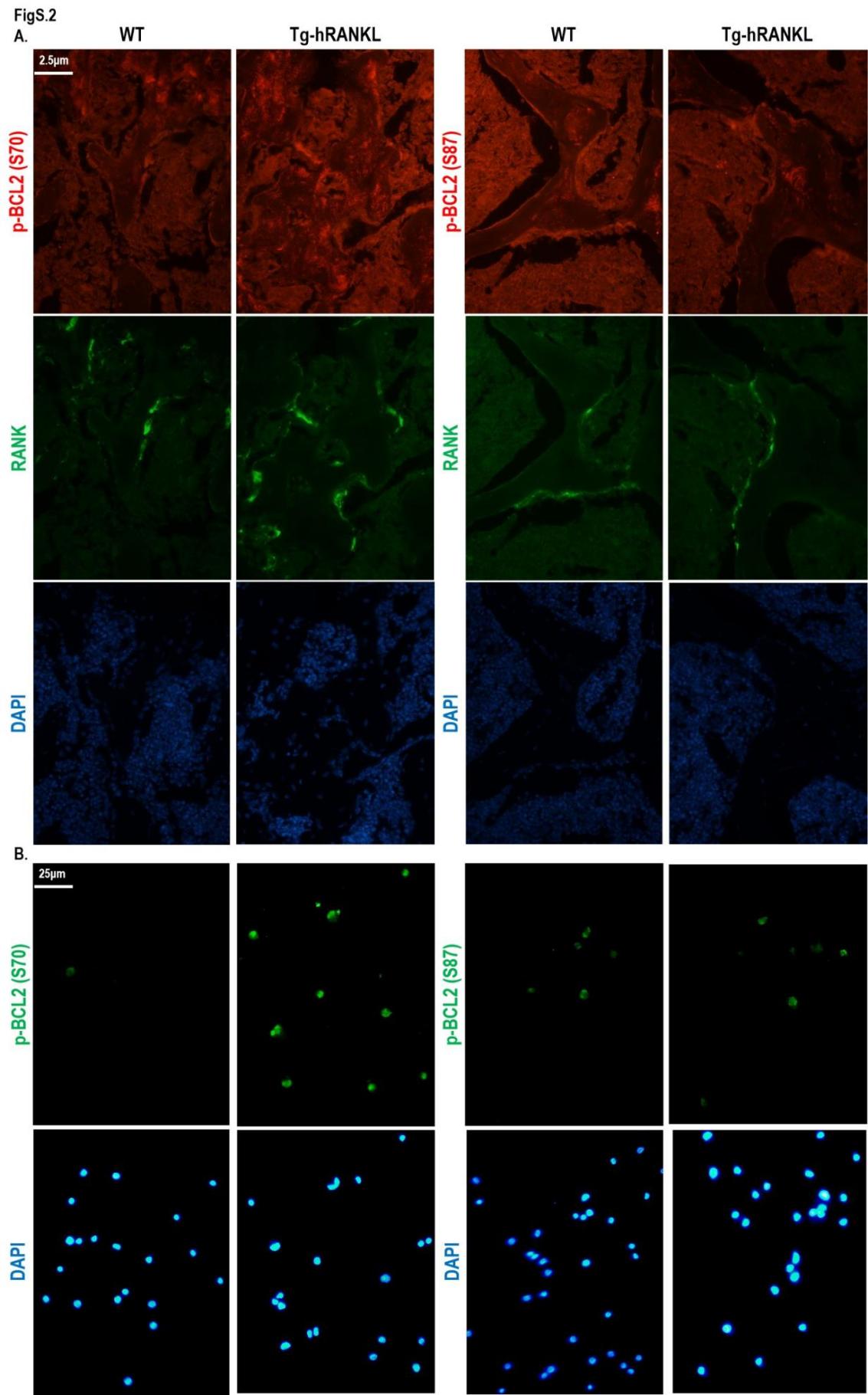


**Fig. S2. Supplementary data on BCL2 phosphorylation in Tg-hRANKL mice.** (A) The single red and green fluorescences for p-BCL2 (S70 or S87) and RANK, respectively, in tibial sections. Scale bar, 2.5 μm. (B) The single fluorescences for p-BCL2 (S70 or S87) and DAPI in bone marrow RANK^+^ CSF1R^+^ cells sorted by FACS. Scale bar, 20 μm. WT, control mice in the same nest.

**
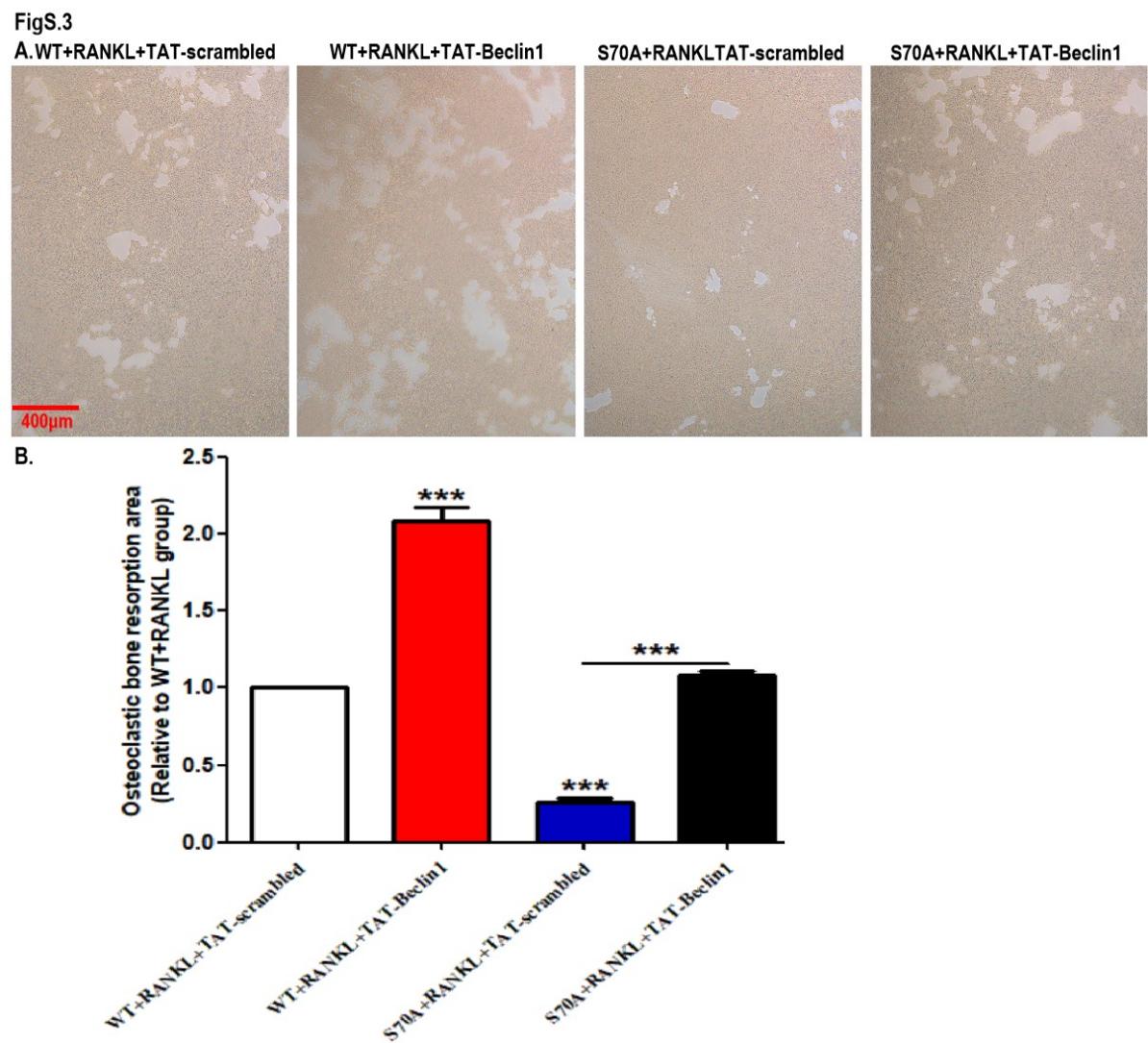
**

**Fig. S3. Bone resorptive activity** **inhibited by BCL2 mutation at S70 was reversed by TAT-Beclin1.** (A) The osteoclastic bone resorptive activity caused by transfected OCPs (transfected with WT BCL2 or S70A BCL2) inoculated on bone discs and treated with M-CSF plus RANKL along with TAT-Beclin1 or TAT-scrambled for 6 days in α-MEM with 5% FBS was evaluated by scanning electron microscopy. Scale bar, 400 μm. (B) Quantitative results showing the mean resorption pit area in **A**. The resorption pit area was represented as normalized to that of control OCPs (WT+RANKL+TAT-scrambled group). The experiments were replicated at least three times. Data are presented as the mean±SEM from three independent experiments. ***P<0.001.
